# Wide Antral Circumferential Re-Ablation for Recurrent Atrial Fibrillation after Prior Pulmonary Vein Isolation Guided by High-Density Mapping Increases Freedom from Atrial Arrhythmias

**DOI:** 10.3390/jcm12154982

**Published:** 2023-07-28

**Authors:** Stefan Hartl, Hisaki Makimoto, Shqipe Gerguri, Lukas Clasen, Sophia Kluge, Christoph Brinkmeyer, Jan Schmidt, Obaida Rana, Malte Kelm, Alexandru Bejinariu

**Affiliations:** 1 Department of Cardiology, Pulmonology and Vascular Medicine, Medical Faculty, Heinrich Heine University, 40225 Düsseldorf, Germany; 2 Department of Electrophysiology, Alfried Krupp Hospital, 45131 Essen, Germany; 3 Department of Medicine, Witten/Herdecke University, 58455 Witten, Germany; 4 Data Science Center/Cardiovascular Center, Jichi Medical University, Shimotsuke 329-0431, Japan; 5 Department of Cardiology, Rhythmology and Angiology, Josephs-Hospital Warendorf Academic Teaching Hospital, University of Münster, 48149 Warendorf, Germany; 6 Cardiovascular Research Institute Düsseldorf (CARID), Medical Faculty, Heinrich Heine University, 40225 Düsseldorf, Germany

**Keywords:** atrial fibrillation, pulmonary vein isolation, wide antral circumferential ablation, re-ablation

## Abstract

Performing repeated pulmonary vein isolation (re-PVI) after recurrent atrial fibrillation (AF) following prior PVI is a standard procedure. However, no consensus exists regarding the most effective approach in redo procedures. We assessed the efficacy of re-PVI using wide antral circumferential re-ablation (WACA) supported by high-density electroanatomical mapping (HDM) as compared to conventional re-PVI. Consecutive patients with AF recurrences showing true PV reconnection (residual intra-PV and PV antral electrical potentials within the initial ablation line) or exclusive PV antral potentials (without intra-PV potentials) in the redo procedure were prospectively enrolled and received HDM-guided WACA (Re-WACA group). Conventional re-PVI patients treated using pure ostial gap ablation guided by a circular mapping catheter served as a historical control (Re-PVI group). Patients with durable PVI and no antral PV potentials were excluded. Arrhythmia recurrences ≥30 s were calculated as recurrences. In total, 114 patients were investigated (Re-WACA: *n* = 56, 68 ± 10 years, Re-PVI: *n* = 58, 65 ± 10 years). There were no significant differences in clinical characteristics including the AF type or the number of previous PVIs. In the Re-WACA group, 11% of patients showed electrical potentials only in the antrum but not inside any PV. At 402 ± 71 days of follow-up, the estimated freedom from arrhythmia was 89% in the Re-WACA group and 69% in the Re-PVI group (*p* = 0.01). Re-WACA independently predicted arrhythmia-free survival (HR = 0.39, 95% CI 0.16–0.93, *p* = 0.03), whereas two previous PVI procedures predicted recurrences (HR = 2.35, 95% CI 1.20–4.46, *p* = 0.01). The Re-WACA strategy guided by HDM significantly improved arrhythmia-free survival as compared to conventional ostial re-PVI. Residual PV antral potentials after prior PVI are frequent and can be easily visualized by HDM.

## 1. Introduction

Worldwide, radiofrequency (RF) catheter ablation is the most frequently performed technique to achieve pulmonary vein isolation (PVI), which equals the procedural endpoint in the primary interventional treatment approach in atrial fibrillation (AF) patients [1]. PVI for rhythm control has been shown to be superior to antiarrhythmic drugs and has demonstrated prognostic benefits in all patient collectives ranging from the asymptomatic AF patient to the AF patient with heart failure [2,3,4,5,6]. Continuous developments of RF technologies, particularly the use of contact-force sensing catheters, as well as standardization of lesion generation using the ablation index (“CLOSE” protocol) have led to an improved ablation line with a reduction in lesion gaps, and thus an increased rate of durable PVI [7]. However, PV reconnections still occur and necessitate redo procedures in many patients. To date, no standardized recommendation exists for patients who require redo procedures, e.g., re-PVI only or re-PVI plus additional ablation. Currently, re-isolation of re-conducted PV is the standard of care in most electrophysiology centers, and ostial gap ablation is frequently performed without proving satisfactory results [8,9]. “PVI plus” strategies involve the re-isolation of reconnected PVs and/or additional ablation of arrhythmogenic substrate, ablation of potential AF trigger regions and empirical lines, ablation of rotors, and empirical left atrial posterior wall ablation. For the latter, a class IIb recommendation exists in an expert consensus statement for redo procedures [10], but recent results have not been entirely convincing [11]. In the initial PVI procedure, wide antral circumferential ablation (WACA) demonstrated superior outcome results as compared to segmental PVI [12]. This can be explained by the fact that the PV antrum is a known arrhythmogenic trigger zone [13] that is additionally ablated by the creation of a wide antral line for PVI. Thus, we hypothesized that WACA re-ablation (Re-WACA) can be a superior strategy as compared to a simple PV targeted re-PVI approach.

The aim of this single-center study was to evaluate the benefit of WACA PV re-ablation (Re-WACA) guided by three-dimensional (3D) high-density left atrial mapping (HDM) as compared to simple ostial re-PVI guided by a circular mapping catheter in patients who experienced AF recurrences due to true PV reconnection or an exclusive documentation of residual antral PV potentials within the initial ablation line (without intra-PV potentials) after previous PVI.

## 2. Materials and Methods

### 2.1. Study Population

The Re-WACA group consisted of unselected, consecutive patients who were scheduled for a redo procedure and underwent WACA re-ablation guided by 3D HDM. Re-WACA patients were prospectively enrolled. Unselected patients who received re-PVI by means of conventional gap ablation at the PV ostium (2016–2018) served as a historical control group and were retrospectively analyzed. Data of the control group were not part of previous studies. The indications for a redo procedure were the same for both groups and corresponded to the inclusion criteria. The inclusion criteria were symptomatic paroxysmal or persistent AF recurrences after prior PVI following a 3 months blanking period. The exclusion criteria were longstanding persistent AF, intracardiac thrombi, and severely dilated left atria (>60 mm). Patients who presented with AT recurrences, patients who showed durable isolation of all PV and/or the PV antrum during the redo procedure, and patients with more than two prior PVI procedures were also excluded.

The power calculation was based on the atrial arrhythmia recurrence rate after standard re-PVI, which was as high as 40% in 12 months [14,15], and, for the Re-WACA group, we hypothesized an arrhythmia recurrence rate of 15% in 12 months (absolute risk reduction of 25%). Considering the follow-up time of more than 12 months in this study, we expected a recurrence rate of 50% and 18.75%, respectively. Hence, a recruitment of 50 patients in the Re-PVI group and 40 patients in the Re-WACA group achieved a statistical power of 0.9. We aimed at a recruitment of at least 50 patients in each group.

### 2.2. Ablation Procedure Protocol

A detailed summary of our standardized RF ablation procedure is available in references [16,17]. Transesophageal echocardiography was performed to rule out intracardiac thrombi. The ablation procedure was conducted under deep analgosedation. Via a triple venous access, a 6F multi-electrode catheter was positioned in the coronary sinus and two SL1 sheaths (St. Jude Medical/Abbott, Plymouth, MN, USA) were advanced into the left atrium following a double fluoroscopy- and pressure-guided transseptal puncture. We aimed at an activated clotting time > 300 s by the administration of intravenous heparin. The PV ostia were visualized by selective PV angiography in combination with 3D mapping. Electrical cardioversion was performed prior to mapping in patients who presented with AF.

In the Re-WACA group, a high-density voltage map of the LA was accomplished by a 3D mapping system (CARTO, Biosense Webster, Inc., Irivine, CA, USA) using a multipolar diagnostic catheter (Pentaray^®^, Biosense Webster, Inc., Irvine, CA, USA). To identify PV reconnections/lesion gaps by displaying the border zone between healthy and scar tissue, the color display range was set to 0.10–0.50 mV. WACA lines were defined according to previous publications [12], e.g., 1.5 cm proximal from the PV ostium. PV reconnection was defined as residual electrical potentials located distally from the defined PV ostium. Antral potentials were defined as residual electrical potentials located within the initial ablation lines but outside of the PV ostium.

Re-WACA by means of contact-force (CF-) guided point-by-point RF ablation was performed in case of (a) true PV reconnection with documented intra-PV and PV antral electrical potentials within the defined WACA line or (b) exclusive documentation of residual PV antral potentials (without the presence of intra-PV potentials).

In the historical Re-PVI control group, an anatomical 3D reconstruction of the left atrium was built (CARTO, Biosense Webster, Inc., Irivine, CA, USA) and lesion gaps were detected by conventional mapping using a circumferential multipolar diagnostic catheter (LASSO^®^, Biosense Webster, Inc., Irvine, CA, USA) and the ablation catheter. Pure ostial gap ablation was performed to re-achieve PVI.

In both study groups, ablation was performed using an irrigated quadripolar ablation catheter with a 3.5 mm tip (ThermoCool^®^ SmartTouch™, Biosense Webster, Inc., Irvine, CA, USA). To achieve durable lesions, a contact force of ≥10 g and a 30 s RF application per point was applied (max. 50 W anteriorly, max. 30 W posteriorly) as the institutional standard at the time [16]. The ablation protocol was adapted early in 2018 due to technological progress, e.g., ablation index (AI)-guided ablation [18], mainly concerning the Re-WACA group. We aimed at an AI of 350 for posterior ablation and 550 for anterior ablation. PVI was the procedural endpoint confirmed by an entrance and exit block following a waiting period of at least 20 min after the last RF application. In both groups, atrial burst stimulation from the coronary sinus was performed (minimal cycle length = 200 ms). If AT was induced or occurred spontaneously, an activation map was generated to understand the arrhythmia mechanism and guide additional ablation. Bidirectional block was confirmed for all ablation lines. The venous access site was supplied with a figure-of-eight suture and a pressure dressing. A fluoroscopy integrating system was implemented during the time course of the study to reduce radiation exposure [19,20].

### 2.3. Post Procedure Observation and Follow-Up

Echocardiography was performed to rule out pericardial effusion immediately after the procedure and again before resuming same day anticoagulation. All patients were observed on the cardiovascular wards with a continuous vital sign and rhythm monitoring for 24 h until routine discharge on the day after the procedure. All complications were documented prospectively. Major complications were referred to as a permanent injury or death, the necessity of interventional treatment, or a prolonged hospital stay [21]. Antiarrhythmic drugs (class IC and III) were discontinued after a 3 month blanking period when stable sinus rhythm was present. Routine rhythm follow-up data were collected at 3 and 12 months (3–7 day Holter monitoring) in the university outpatient clinic and more frequently in case of AF symptoms. Atrial arrhythmias lasting for at least 30 s were calculated as recurrences. Consultations with collaborating centers and telephonic interviews with patients completed the follow-up.

### 2.4. Statistical Analysis

Continuous data are displayed as mean with standard deviation (SD) or median with interquartile range in the case of a non-normal distribution, and were compared using the Student’s *t*-test or the non-parametric Mann–Whitney U test. Categorical data are presented as numbers and percentages, and were compared utilizing the chi-square or the Fisher’s exact test, respectively. Estimated event-free survival was calculated and displayed using the Kaplan–Meier method. Cox regression analyses identified predictors for recurrences. A *p*-value < 0.05 was considered to be statistically significant. Data processing and analysis were performed using SPSS (IBM SPSS Statistics, Version 22.0, Armonk, NY, USA).

## 3. Results

### 3.1. Baseline Characteristics

All baseline characteristics are summarized in Table 1. In total, 114 patients with symptomatic AF recurrences after previous PVI underwent re-ablation. Fifty-six patients of the Re-WACA group, 54% male, with a mean age of 68 ± 10.0 years and a median CHA_2_DS_2_-VASc score of 3, suffered from paroxysmal AF (46%) compared to 58 patients of the Re-PVI control group (69% male, 64.9 ± 10.3 years, median CHA_2_DS_2_-VASc score 2, 34% paroxysmal AF). Patients in the Re-WACA group tended to be older (*p* = 0.08) and ischemic heart disease was more frequently observed (*p* = 0.05), whereas all other baseline characteristics did not show significant differences between the groups including the AF type, the LA size, and the number of previous LA ablation procedures.

### 3.2. Procedural Data

All procedural data are summarized in Table 2. During the redo procedure, the number of non-isolated PVs was documented in both groups, estimating 2.4 ± 1.1 in the Re-WACA group and 2.7 ± 1.0 in the Re-PVI group (*p* = 0.15). Right-sided PVs were both more frequently non-isolated in the Re-PVI group (*p* = 0.03 for the RIPV and *p* = 0.01 for the RSPV, respectively). In the Re-WACA group, 89% of the patients demonstrated potentials inside the PV implicating true PV reconnection. Any non-isolation of the PV antrum was present in all patients: 71% of patients showed residual right-sided PV and 79% of patients showed residual left-sided PV antral potentials. In 50% of the patients, PV antral potentials were noticed in both right- and left-sided PVs. In 11% (*n* = 6) of the Re-WACA group, only antral electrical potentials within the initial ablation line were noted, but no electrical potentials were seen inside the PVs. This finding was not associated with AT supporting that potential source of origin. All of these six patients received prior cryoablation and one patient received prior cryo- and RF ablation. In the Re-PVI group, PV reconnection with a documentation of PV potentials was present in all patients. In both groups, all PV were successfully re-isolated. Additional LA ablation for induced or spontaneous arrhythmias (Table 2) was performed in both groups as required, which was more frequently the case in the Re-PVI group (*p* < 0.01). The adverse event rate was 3.5% (*n* = 4/114) in the entire study population and did not differ between the groups (*p* = 1.0). Major periprocedural complications occurred in 1.8% including one pericardial tamponade requiring drainage and one small intracranial bleeding that could be managed conservatively (Table 3). No permanent sequelae or death occurred.

### 3.3. Follow-Up

**Figure 1:**
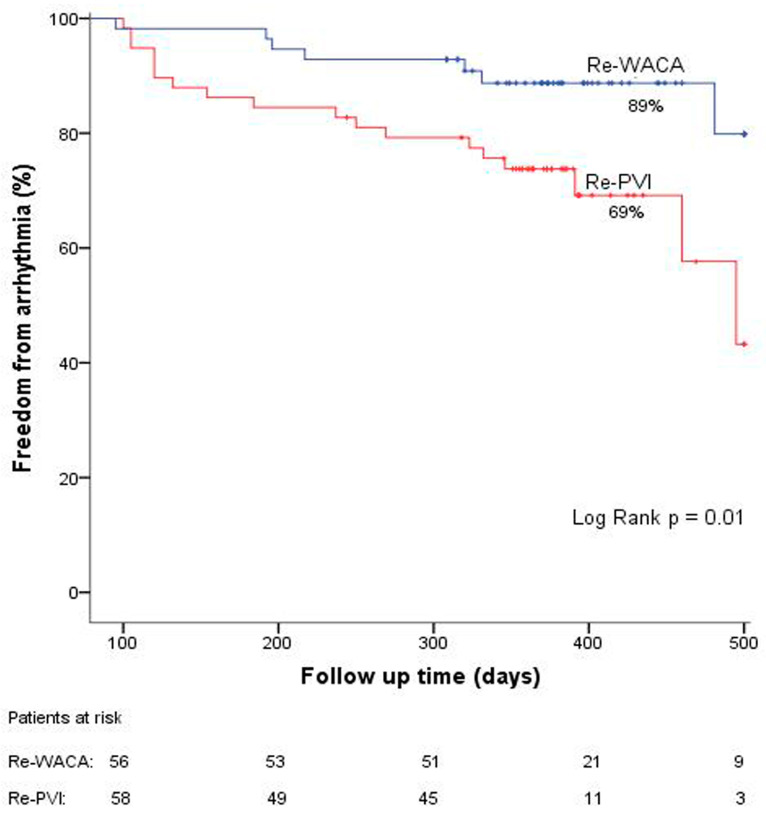
Kaplan–Meier estimates of freedom from arrhythmia recurrences. PVI, pulmonary vein isolation; WACA, wide area circumferential ablation.

After a mean follow-up of 402 ± 71 days, the Kaplan–Meier estimate (Figure 1) demonstrates significant advantages of the Re-WACA strategy as compared to the Re-PVI approach: Freedom from arrhythmia was 89% and 69%, respectively (*p* = 0.01). In total, 13 (*n* = 7/56 patients) of the Re-WACA group and 31% (*n* = 18/58 patients) of the Re-PVI group experienced arrhythmia recurrences (*p* = 0.02). The mean time to arrhythmia recurrence was 259 ± 130 days and 227 ± 139 days, respectively. At follow-up, 4 of initially 35 patients of the entire study group were on antiarrhythmic drugs. A Cox regression analysis (Table 4) revealed that the Re-WACA strategy independently predicted freedom from atrial arrhythmia (HR = 0.39, 95% CI 0.16–0.93, *p* = 0.03), whereas two previous PVI procedures predicted recurrences (HR = 2.35, 95% CI 1.20–4.46, *p* = 0.01).

## 4. Discussion

This study was conducted to investigate the potential benefit of PV re-isolation using wide antral circumferential re-ablation guided by 3D electroanatomical HDM as compared to conventional re-PVI by means of pure ostial gap ablation in patients who experienced symptomatic AF after previous PVI. Patients with true PV re-conduction who demonstrated intra-PV and PV antral potentials within the initial ablation zone or patients with exclusive residual PV antral potentials (without showing intra-PV potentials) were included. The key findings are: (1) HDM demonstrated a high incidence of residual PV antral potentials, even in cases in which PVs were isolated. (2) WACA re-ablation is significantly effective to reduce arrhythmia recurrences as compared to conventional re-PVI without HDM (Figure 1).

It is well known that durable PVI is important regarding clinical success after interventional treatment of AF. PV reconnections could be substantially reduced after major technical improvements in RF-ablation within recent years, e.g., the implementation of contact-force sensing catheters, the ablation index [7,10], or advanced cryoballoon generations [22,23]. However, PV reconnections are still common and represent the predominant mechanism of AF recurrences [24,25]. Repeated PVI has demonstrated an improvement in arrhythmia free survival [8]. Nevertheless, approximately one fifth of patients experience recurrences despite durable PVI, which leads the focus on whether additional ablation can improve rhythm outcome. So far, “PVI plus” strategies in index or redo procedures have not yieldedconsistent results [14,26,27,28,29], and extensive (incomplete) ablation can increase the incidence of iatrogenic atrial arrhythmias and procedural complications [30]. To date, no consensus exists regarding the optimal ablation strategy in redo procedures.

The atrial-PV junction is an arrhythmogenic area. Next to PV foci, the area consists of a complex myocardial fiber orientation with abrupt changes in direction as shown in animal models [31,32,33] and human individuals [34,35,36], which can lead to conduction abnormalities and facilitate arrhythmogenesis. Gottlieb et al. recently concluded that triggers of AF likely involve re-entry mechanisms in the atrial-PV junctional myocardium [13]. Additionally, endo-epicardial connections arising from the PV antrum have been reported [37,38] and could be relevant in terms of residual antral potentials within the initial ablation area, as seen in our cohort. The PV antrum includes parts of the intrinsic cardiac autonomic systems, e.g., ganglionated plexi that have also been targeted to improve the outcome of AF ablation [39] and other non-PV substrates resulting from infiltration of adipose tissue and collagen accumulation due to AF itself [40] leading to fibrosis [41]. Kiuchi et. al showed that a larger isolation area around the PV was associated with reduced arrhythmia recurrences and they stated that the main mechanism of recurrence was considered to be a residual substrate in the PV antrum [42]. Furthermore, when performing wide antral ablation, parts of the posterior LA wall, which has the same embryological origin as the PVs and is thought to play a crucial role in the initiation and maintenance of AF [43], are empirically ablated.

In this study, patients of the Re-WACA group who were investigated by HDM guidance showed PV antral potentials in all patients, even in those without intra-PV potentials. Re-WACA patients significantly benefited from a wide antral circumferential re-ablation as compared to the Re-PVI group and showed a similar low adverse event profile. The Re-WACA strategy independently predicted freedom from arrhythmias, whereas more than one previous procedure predicted recurrences. Patients showing solely antral potentials were all treated by cryoablation before, which supports previous findings [44]. However, no association with AT regarding that potential source of mechanism could be drawn from our data. Notably, additional lesion sets beyond PVI were performed significantly more often in the Re-PVI group (25% vs. 67%, *p* < 0.01) due to spontaneous or induced AT, but did not translate into an improved outcome. Our findings highlight the importance of HDM and its technical advantages over conventional mapping for the detection of arrhythmogenic foci in terms of inhomogenity or even non-transmurality within the initial lesion set, and the superior clinical outcome of the Re-WACA strategy emphasizes the arrhythmogenic potential of the PV antral area in general. The clinical relevance of our data is high, since redo procedures after PVI using single shot technologies such as cryoballoon ablation and pulsed field ablation [45] will dramatically increase with the inherent need for deployment of more antral lesions. Future implementation of artificial intelligence could optimize ablation strategies and improve outcome further [46].

Based on our data, the Re-WACA strategy can be a treatment option for redo procedures in patients with symptomatic AF recurrences and non-isolated PV or residual antral potentials. However, further data of larger patient populations are necessary to confirm our findings.

There are several limitations. This study is a non-randomized single center study with consecutive patients being compared to a historical control group. In the control group (Re-PVI without HDM), the documentation of antral PV potentials was not assessed or might have been underestimated due to technical limitations of conventional mapping [47]. Hence, a comparison of the incidence of antral PV potentials was not possible and the significance of low voltage areas in the LA, that can be relevant for ablation strategies [48], could not be reliably assessed. CF measurements were available for both study groups. However, AI-guided ablation became available for the majority of patients of the Re-WACA group during the study period, which could have influenced outcome results. However, since our previous ablation settings mostly correspond to AI-guided ablation, we assume only a negligible bias. Additionally, both paroxysmal and persistent AF patients were included and up to two prior PVI procedures were allowed for inclusion, which might have affected the outcome results; however, the sample size allowed for statistical control of such characteristics. Finally, no continuous rhythm monitoring was available, which could overestimate outcome results.

## 5. Conclusions

Patients with recurrent AF after initially successful PVI show a high incidence of residual PV antral potentials in the redo procedure. Wide antral circumferential PV re-ablation guided by 3D electroanatomical HDM was significantly effective to reduce arrhythmia recurrences without compromising safety as compared to conventional re-PVI by pure ostial gap ablation. The re-WACA strategy can be a treatment approach in patients who show reconnected PVs or exclusive residual antral PV potentials in the redo procedure.

## Figures and Tables

**Table 1:** Baseline characteristics.

	Re-WACA *n* = 56	Re-PVI *n* = 58	*p-*Value
Age (yrs)	68.3 ± 10.0	64.9 ± 10.3	0.08
Male sex, *n* (%)	30 (54)	40 (69)	0.09
CHA_2_DS_2_-VASc Score	3 [2–4]	2 [1–4]	0.20
Arterial Hypertension, *n* (%)	43 (77)	40 (69)	0.35
Diabetes mellitus, *n* (%)	7 (13)	11 (19)	0.34
Ischemic heart disease, *n* (%)	20 (36)	11 (19)	0.05
COLD, *n* (%)	4 (7)	2 (3)	0.43
Obstructive sleep apnea, *n* (%)	8 (14)	12 (22)	0.26
Body Mass Index, kg/m^2^	26.0 ± 5.7	27.2 ± 4.2	0.22
LVEF, (%)	58.5 ± 10.0	59.8 ± 9.6	0.48
Serum Creatinine, mg/dL	0.97 ± 0.27	1.05 ± 0.29	0.11
NTproBNP, pg/mL	581 [219–977]	366 [175–947]	0.31
Dyslipidemia, *n* (%)	27 (48)	30 (52)	0.71
LA Diameter, cm	4.2 ± 1.0	4.1 ± 0.8	0.77
Months since AF diagnosis	48 [22–75]	48 [11–72]	0.59
Number of previous PVI, *n*	1.27 ± 0.52	1.43 ± 0.53	0.10
Technique of prior PVI			
- RF, n (%)	24 (43)	27 (47)	0.69
- Cryoballoon, *n* (%)	18 (32)	9 (16)	0.04
- unknown n (%)	5 (9)	12 (21)	0.08
- multiple, n (%)	9 (16)	10 (17)	0.87
Months since last PVI	17 [9–42]	18 [4–56]	0.43
Indication for redo procedure			
- Paroxysmal AF, *n* (%)	26 (46)	20 (34)	0.19
- Persistent AF, *n* (%)	30 (54)	38 (66)	
EHRA Score (2/3/4), *n*	4/47/5	4/46/8	0.72
Beta-Blockers, *n* (%)	46 (82)	40 (70)	0.14
AAD, *n* (%)	12 (21)	23 (40)	0.03

Data are presented as *n* (%) of patients, mean ± SD, or median [IQR]. COLD, chronic obstructive lung disease; LVEF, left ventricular ejection fraction; LA, left atrium; PVI, pulmonary vein isolation; RF, radiofrequency; AAD, antiarrhythmic drug (class IC or III); WACA, wide area circumferential ablation; EHRA, European Heart Rhythm Association, AF, atrial fibrillation.

**Table 2:** Procedural data.

	Re-WACA *n* = 56	Re-PVI *n* = 58	*p-*Value
Procedure time (min)	135 ± 42	142 ± 47	0.39
Fluoroscopy time (s)	828 ± 298	726 ± 258	0.05
Dose Area Product (cGy cm^2^)	901 ± 678 *	2048 ± 1111	<0.01
Number of non-isolated PV	2.4 ± 1.1	2.7 ± 1.0	0.15
- non-isolated RSPV, *n* (%)	26 (46)	39 (67)	0.03
- non-isolated RIPV, *n* (%)	31 (55)	45 (78)	0.01
- non-isolated RPV antrum, *n* (%)	40 (71)	n.a.	
- non-isolated LSPV, *n* (%)	33 (59)	37 (64)	0.59
- non-isolated LIPV, *n* (%)	35 (63)	37 (64)	0.89
- non-isolated LPV antrum, *n* (%)	44 (79)	n.a.	
- any non-isolated PV antrum, *n* (%)	56 (100)	n.a.	
No potentials inside any PV, *n* (%)	6 (11)	0 (0)	0.01
Additional LA ablation, *n* (%)	14 (25)	39 (67)	<0.01
- Anterior line	8 (14)	17 (29)	0.05
- Roof line	3 (5)	11 (19)	0.03
- Mitral isthmus line	1 (2)	3 (5)	0.33
- CFAE ablation	2 (4)	8 (14)	0.05

Data are presented as mean ± SD or *n* (%). WACA, wide area circumferential ablation; PVI, pulmonary vein isolation; RSPV, right superior pulmonary vein; RIPV, right inferior PV; RPV, right PV; LSPV, left superior PV; LIPV, left inferior PV; LPV, left PV; LA, left atrium; CFAE, complex fractionated atrial electrograms. * Dose area product was reduced due to a new fluoroscopic system [19,20].

**Table 3:** Adverse event rate.

	Re-WACA *n* = 56	Re-PVI *n* = 58	*p-*Value
Major complications, *n* (%)	1 (1.8)	1 (1.7)	1.00
- Pericardial tamponade, *n*	1	0	0.31
- Gastroparesis, *n*	0	0	
- Atrioesophagela fistula, *n*	0	0	
- TIA/Stroke *n*	0	0	
- Intracranial bleeding, *n*	0	1	0.32
- Death, *n*	0	0	
Minor complications, *n* (%)	1 (1.8)	1 (1.7)	1.00
- Pericarditis, *n*	0	0	
- Groin hematoma, *n*	1	1	
- Infections, *n*	0	0	
Overall complications, *n* (%)	4 (3.5)		

Data are presented as *n* (%) or *n*. WACA, wide area circumferential ablation; PVI, pulmonary vein isolation; TIA, transient ischemic attack.

**Table 4:** Cox regression analysis to identify predictors for arrhythmia recurrences.

	Univariate Analysis			Multivariate Analysis		
	HR	95% CI	*p*-Value	HR	95% CI	*p*-Value
Number of previous PVI (*n* > 1)	2.50	1.33–4.52	<0.01	2.35	1.20–4.46	0.01
Re-WACA	0.33	0.14–0.80	0.01	0.39	0.16–0.93	0.03
Number of non-isolated PVs	0.77	0.52–1.12	0.18			
Age	0.99	0.96–1.03	0.70			
Male sex	0.80	0.36–1.77	0.59			
CHA_2_DS_2_-VASc Score (1 point)	1.05	0.83–1.29	0.69			
LV-EF (1%)	0.97	0.94–1.01	0.14			
LA diameter (1 cm)	1.26	0.84–1.88	0.26			
Obstructive sleep apnea	1.49	0.59–3.73	0.40			
BMI (1 kg/m^2^)	1.01	0.93–1.09	0.75			

PV(I), pulmonary vein (isolation); LV-EF, left ventricular ejection fraction; LA, left atrium; BMI, body mass index; WACA, wide area circumferential ablation; CI, confidence interval.

## Data Availability

Raw data were generated at the University Hospital in Düsseldorf, Germany. The data that support the findings of this study are available from the principal investigator on reasonable request.
